# Amebic Catarrh of the Intestinal Tract1Read at the twenty-eighth annual meeting of the Medical Association of Central New York, at Syracuse, October 15, 1895.

**Published:** 1896-05

**Authors:** A. A. Young

**Affiliations:** Newark, N. Y.


// BUFFALO MEDICAL JOURNAL.

VOL. XXXV. MAY, 1896. No. 10.

### Original Communications.

### AMEBIC CATARRH OF THE INTESTINAL TRACT.[1]

BY A. A. YOUNG, M. D., Newark, N. Y.

IN OCTOBER, 1892, I was called to see a lad, about 10 years of age, having a temperature of 105½° F., together with a slight diarrhea. The discharges were of a brownish-red color, thin and scanty. His tongue was slightly furred and of a brownish appearance. There were no other apparent or marked symptoms.

At my second visit, on the following day, I found no change of condition, and the inference was then drawn that I had, so far as I was concerned, a new affection to deal with. A consultation was had with my library, and from that I turned away none the wiser. A further investigation seemed imperative and with the appliances at hand the task was begun.

At the bottom of the vessel containing the fecal discharges were small masses of matter which macroscopically had the appearance of berry seeds. These I believed to be the key to the situation. An examination by the microscope showed them to be not berry seeds at all, but an aggregation of cell-growths, each having an independent life, each capable of producing growths of its kind. In size the individual cell was perhaps three or four times as large as the white blood corpuscle, oval in form and, so far as could be ascertained, developed by the process of budding. Accepting the theory that on the development of these germs the disease depended, various intestinal antiseptics were prescribed, but without much marked relief. After about three weeks Dr. John Van Duyn, of Syracuse, N. Y., was consulted. He ventured the opinion, based upon my report, as to the disease being one of intestinal sepsis, primary cause a questionable agent. As to the treatment, no change was advised, which consisted in the administration of salol, salicylate of bismuth, fel porci and olive oil, together with copious and frequent enemas impregnated with creolin were used. Foods were discontinued as far as possible, save water, which was given in

1. Read at the twenty-eighth annual meeting of the Medical Association of Central New York, at Syracuse, October 15, 1895.

abundance. Liquid foods were only allowed and those foods devoid of starch or sugar prescribed.

After about five weeks of what appeared to be a tedious and fruitless service, passages were obtained of a mushy character and of a greenish-black color. These passages proved to be, largely, vitiated bile filled with the cell-growths above referred to. After this passage, signs of improvement were noted, although convalescence was slow and tedious, requiring several months to regain his ordinary strength. In want of a better term, then, for this condition, although the name is doubtless a misnomer, the disease was denominated sporadic intestinal catarrh.

From investigation it was evident to me that the presence of these cells (spores) or amebæ were either due to putrefactive changes in the food products in the alimentary canal or degenerative changes in some of the digestive excretions.

To classify the signs and symptoms of this affection is far beyond my skill, so varied were they. The only evidences of this disease common to all patients, as I have observed them, were anorexia, tenderness (but not marked) along the inferior border of the liver, stools scanty and of a reddish-brown color containing a large number of peculiar cell-growths or amebæ. Urine scanty, high colored and containing an abnormal amount of uric acid. Other signs and symptoms varied from one extreme to the other. The temperature in some cases was as low as 96° F., while in others it reached the 106° F. mark. In some cases pain was intense and not limited to any particular portion of the body, while in others there was a complete absence of pain; the patient simply desired to be let alone in every particular. In some cases the pulse would rise to 140 or more, while in other cases a pulse of 30 or 40 was noted. There also seemed to be a coincidence between a slow pulse and absence of pain. Where there was a pulse of 60 or lower, I remember of no case suffering any pain. This phenomenon I am not prepared at this time to discuss.

From the bronzed condition of the skin, appearance of the stools, and the like, I was led to infer that this affection is not primarily a disease of the alimentary canal or any portion of it, but one that has its beginnings within the ducts of the liver, and that the presence of the germs within the bowel is a coincidence or an accident. To show that their true nidus is within ducts of the liver and that their favorite developing media is the bile, whether vitiated or normal, is one of the objects of this paper. The general symptomatology, further than has been stated, will therefore be passed by, and in lieu thereof your attention called to a most excellent article, entitled Amebic dysentery, by Dr. Charles G. Stockton, of Buffalo, N. Y., in Volume I., fourth series, *International Clinics.* The affection there described is doubtless the same as the one under consideration and of which our text-books are conspicuously silent.

As has been already intimated, the greenish-black stool, which was composed of vitiated bile and amebæ, always preceded recovery, and the advent of such stools marked the turning point and was an index that convalescence was near. This phenomenon, so patent to me, gave a forcible impression that the liver was the organ in which the pathological condition first made its appearance and that all other allied pathological conditions outside of this organ were secondary or the sequelæ from the invasion of the liver. This theory was materially strengthened by being present at an autopsy, held December 26, 1892, under the auspices of another physician. The previous history of this case, as given by members of the family and attending physician, left no room for doubt but that the affection under consideration (called by one of the attending physicians "winter cholera"), but really amebic dysentery (Stockton), was the cause of his death.

The autopsy revealed pathological conditions existing in the liver and the intestinal tract and all other organs apparently normal. The ducts of the liver were distended and filled with vitiated bile, which was infested by large numbers of amebæ. The liver was abnormally dark and somewhat enlarged; the inferior border extending to one inch below the border of the ribs. The stomach was normal, but below this organ the entire alimentary canal was involved. The intestines contained more or less fecal matter of a reddish-brown color, and of a semi-solid consistency and also containing amebæ in large numbers.

The internal membrane of the bowels was in a semi-disintegrated condition and had evidently lost its integrity previous to death. There was no obstruction of the common gall duct, or other ducts of the liver, save that produced by the gall itself, which was in a gelatinised condition, a condition which I then believed, and still believe, was produced by the amebæ. Suffice it to say that the ante mortem appearance of this patient was similar to that which we have in obstruction of the gall-duct in a mechanical way. This condition appeared about one week previous to death.

Another case still more marked and from which more positive inferences were drawn, came under my observation April 18, 1894, a brief history of which will here be given:

N. M., of Arcadia, N. Y., aged about 65, called upon me, stating that on or about January 1, 1894, he was attacked with a mild diarrhea, unaccompanied by pain, and that the stools were in a slimy condition and of a reddish-brown color, changing occasionally to a gray color. Examination of the stools revealed the presence of amebæ. His tongue was slightly coated, largely on posterior half, and red along the free borders. There was no tenderness in the epigastrium, and some slight pains at times, especially after partaking of food, near the umbilicus. He did not partake of food on account of hunger, but because he considered it a necessity, a duty. During his entire illness the pulse, temperature and respiration ratio were scarcely changed, remaining at, pulse, 76; temperature, 98½°; respiration, 20, except the latter two weeks of life, when the frequency of the pulse was lowered, varying from 50 to 60. Up to May 15th, no material change in his condition was noted, save the gradual loss of flesh and strength. About this date jaundice appeared and became more and more marked up to the time of his death, which occurred about May 27, 1894.

So far as it was possible to learn, there was no elevation of temperature or any material pain from the onset to the close of the disease. The patient simply asked to be let alone. About ten hours after death an autopsy was held and no abnormal conditions found, save those directly connected with the liver. This organ was about normal in size, but of almost a black color and friable. The gall-bladder was empty and the ducts leading from it free and unobstructed. Beginning just above the juncture of the gall-bladder with the gall-duct, there was marked enlargement of the ducts extending into the body of the liver. These ducts were distended from two to three times their natural size by gelatinised gall, in which there was an abundance of these new growths or amebæ. There was no abnormal condition of the intestinal tract, save atrophy of tissues. This condition, however, I believe was due in a large measure to the nonuse of the organs, for since January 1st he had partaken of but little food, and for the last three weeks of his life practically none. In the intestinal tract there was but very little feculent matter. In this, however, a few amebæ were found, not in sufficient numbers to injure the integrity of the tissues.

There being no other cause for death found, save the pathological condition found in the liver, it is fair to infer that the disease

(which may mean amebic development) began, continued and ended within the hepatic ducts; and if in one case it so develops, may it not be the case in all, and may not the presence of amebæ within the intestines be a coincidence only?

If what I have surmised be true as to the etiology of this disease, then our nomenclature must be changed and with it come a corresponding change of treatment also, a treatment as it now appears that will liquify the gelatinised gall and cause its exit through its proper channel into the receptacle nature has provided for it, and the amebæ which it then contains nature may herself destroy and expel or allow us so to do.

